# NaViA: a program for the visual analysis of complex mass spectra

**DOI:** 10.1093/bioinformatics/btab436

**Published:** 2021-06-19

**Authors:** Daniel Quetschlich, Tim K Esser, Thomas D Newport, Francesco Fiorentino, Denis Shutin, Siyun Chen, Rachel Davis, Silvia Lovera, Idlir Liko, Phillip J Stansfeld, Carol V Robinson

**Affiliations:** Department of Chemistry, University of Oxford, Oxford OX1 3TF, UK; Department of Biochemistry, University of Oxford, Oxford OX1 3QU, UK; Department of Chemistry, University of Oxford, Oxford OX1 3TF, UK; Department of Biochemistry, University of Oxford, Oxford OX1 3QU, UK; Department of Chemistry, University of Oxford, Oxford OX1 3TF, UK; Department of Chemistry, University of Oxford, Oxford OX1 3TF, UK; Department of Chemistry, University of Oxford, Oxford OX1 3TF, UK; UCB Pharma, Slough, Berkshire SL1 3WE, UK; UCB Pharma, Chemin du Foriest, 1420 Braine-l’Alleud, Belgium; OMass Therapeutics, Oxford OX4 4GE, UK; Department of Biochemistry, University of Oxford, Oxford OX1 3QU, UK; School of Life Sciences & Department of Chemistry, University of Warwick, Coventry CV4 7AL, UK; Department of Chemistry, University of Oxford, Oxford OX1 3TF, UK

## Abstract

**Motivation:**

Native mass spectrometry is now a well-established method for the investigation of protein complexes, specifically their subunit stoichiometry and ligand binding properties. Recent advances allowing the analysis of complex mixtures lead to an increasing diversity and complexity in the spectra obtained. These spectra can be time-consuming to tackle through manual assignment and challenging for automated approaches.

**Results:**

Native Mass Spectrometry Visual Analyser is a web-based tool to augment the manual process of peak assignment. In addition to matching masses to the stoichiometry of its component subunits, it allows raw data processing, assignment and annotation and permits mass spectra to be shared with their respective interpretation.

**Availability and implementation:**

NaViA is open-source and can be accessed online under https://navia.ms. The source code and documentation can be accessed at https://github.com/d-que/navia, under the BSD 2-Clause licence.

**Supplementary information:**

Supplementary data are available at *Bioinformatics* online.

## 1 Introduction

Native mass spectrometry (nMS) is an established technique that allows a label-free analysis of intact proteins and their complexes in a mass spectrometer (Marty *et al.*, 2015). It has proven to be a powerful tool in investigating the stoichiometry and interactions of a wide variety of proteins with unprecedented resolution (Bolla *et al.*, 2019). In nMS, protein complexes are transferred into the mass spectrometer by means of electrospray ionization (Dole *et al.*, 1968). A series of ion selection steps and application of energy through accelerating voltages and collisions with inert gas molecules allow the transmission of folded proteins and interaction partners into a mass analyser (Gupta *et al.*, 2018). Soluble proteins and protein complexes with molecular weights up to the mega-Dalton mass range are amenable to study and examples include ribosomes (McKay *et al.*, 2006) and intact viruses (Snijder *et al.*, 2008). Also, more recently membrane proteins have been studied following their release from their solubilizing agent (e.g. detergent micelles, bicelles, nanodiscs or vesicles) through application of energy prior to mass analysis (Laganowsky *et al.*, 2013). Mass spectra are then analysed manually or by using automated approaches such as UniDec (Bern *et al.*, 2018; Marty *et al.*, 2015). Interpretation of these spectra requires a priori knowledge of the molecules included in the complex from gel electrophoresis, western blot or ‘omics techniques (Gault *et al.*, 2016). 

Recent progress in sample preparation paved the way for the investigation of increasingly complex environments such as vesicles obtained from cell membranes. Following this procedure, samples are injected directly into the mass spectrometer and analysed through nMS without previous purification or treatment with detergents (SoLVe-MS) (Chorev *et al.*, 2018), to preserve the native conditions for membrane proteins. These experiments yield highly complicated spectra containing a plethora of charge state series corresponding to the different protein complexes present in the vesicle. Analysing these spectra through high-level automated approaches is challenging given the extent of overlapping peak series. Thus, performing a manual analysis of these spectra is necessary yet incredibly time-consuming.

**Fig 1.**
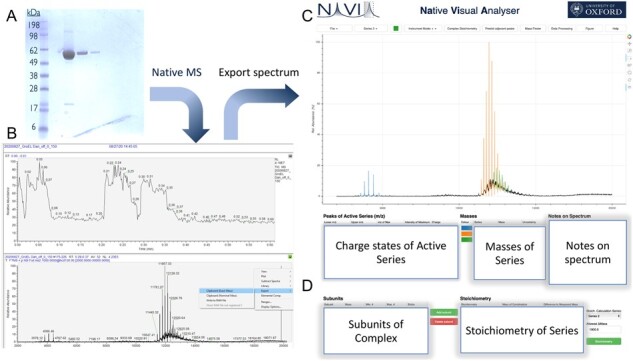
Example of the integration of NaViA into native mass spectrometry workflow. (**A**) In this example purified GroEL purchased from Sigma Aldrich was used as a sample. (**B**) The native mass spectrometry spectrum can be exported as a CSV file. (**C**) Import CSV file into NaViA and analyse by visually selecting peaks for each molecular species. (**D**) Assess stoichiometry for identified species though ‘Complex stoichiometry’ in NaViA

Motivated by these necessities, we created Native Mass Spectrometry Visual Analyser (NaViA), with the aim of occupying the middle ground between manual and automated analysis. Therefore, NaViA is complementary to existing software (e.g. UniDec). NaViA is a web service that allows a semi-automated analysis of native mass spectra through an easy-to-use interface (Fig. 1). Charge states corresponding to a particular protein are selected visually and masses are assigned automatically following known algorithms. The focus on augmenting a manual assignment is also instructive for scientists entering the field of nMS, since it provides key information on where peaks are to be expected for a certain complex and highlights peak series for this complex. In addition, sessions may be saved comprising both raw data and also processed data with annotations. This permits the nMS community to share both, spectra and the analysis, in a standardized format online as part of a collaboration or publication.

## 2 Materials and methods

### 2.1. Implementation

The web service is implemented in Python using the Bokeh visualization package. It is a standalone HTML file without any server in the background. It is implemented in Python 3.6 and JavaScript using the Bokeh framework (version 1.4.0). According to Wellcome and the UK research and innovation’s Common Principles on Data Policy on data, software and materials management and sharing, all data supporting this study will be openly available from the software repository.

### 2.2. Documentation and tutorials

The documentation of NaViA is available on its GitHub repository as well as an introductory video (https://www.youtube.com/channel/UCRvzQxegz0WNkjvvaRJ8PpQ/).

### 2.3. Input

NaViA currently loads files in a CSV format of the two columns *m*/*z* and intensity. These can be exported by commonly used software such as MassLynx for Waters Instruments or Xcalibur for ThermoFisher Scientific instruments such as OrbiTraps (Makarov, 2000). Upon loading, the intensities are normalized. 

## 3 Results

### 3.1 Processing of mass spectra

NaViA enables smoothing and background subtraction of native mass spectra using the same algorithm as standard software, e.g. UniDec. Smoothing is performed through a Gaussian filter and can be performed multiple times. In addition, different modes of background subtraction are facilitated and a filter for ‘minimum intensity’ can be applied. The background subtraction modes include subtracting a fixed value, a linear interpolation between the intensities of the smallest and largest *m*/*z* values and a curved subtraction as described previously (Morgner and Robinson, 2012).

### 3.2 Peak selection and mass calculation

Peaks are selected through a range on the *m*/*z*-axis using the mouse cursor. Once multiple charge states are selected, the corresponding mass is calculated by the MacSED algorithm (McKay *et al.*, 2006). This algorithm systematically creates a matrix of possible charge states assuming there are no missing charge states in the series. Based on this series a matrix of masses is created where each *m*/*z* value of a peak is deconvoluted into a mass value. The variance between the masses is then calculated for each series of charges. The series of masses with the minimal variance is then selected to be the true series of charge states. Finally, the mean of the measured mass is set as the true mass and the standard deviation set to be the uncertainty value.

### 3.3 Tools for augmenting peak assignment

For the analysis of complex native mass spectra two tools have been implemented: a ‘Mass Finder’, which allows the user to highlight *m*/*z* regions for a specific mass of a complex, and a ‘Peak Prediction’ tool, which highlights adjacent peaks for a known series. The ‘Mass Finder’ makes it possible to see whether a complex with known mass is present. This is especially important for SoLVe spectra where many molecular species are present. For these spectra, ‘Peak prediction’ augments the differentiation of overlapping peak series.

### 3.4 Comparing complex mass to subunit masses

The ‘Complex Stoichiometry’ function makes it possible to see whether a complex with known mass is present. This is especially important for cases where many molecular species are present.

### 3.5 Sessions in NaViA

NaViA allows sessions to be saved, including raw data, data processing and peak assignment, in a *navia* file in JSON format. This feature allows users to share spectra including assignments as well as collaborating or deposition of mass spectra for enabling raw data sharing. In addition, JSON formats are easily readable for various programming languages and software packages, e.g. Python or Origin. Therefore, *navia* files are well suited for consecutive data analysis. The format is described in the documentation on the GitHub page.

### 3.6 Compatibility with UniDec

NaViA was developed to be complementary to the current software packages available for native MS data analysis. As part of this, an option to import NaViA sessions has been incorporated into the current UniDec version (4.4.1) (Experimental → Import from NaViA).

### 3.7 Exporting images

NaViA provides the possibility to export images in 4k quality sufficient for A4 pages in publications as well as vector graphics in the SVG format.
